# Developing a value assessment index system of anti-tumour commercial Chinese polyherbal preparation in China: a modified Delphi study

**DOI:** 10.3389/fphar.2025.1681174

**Published:** 2025-12-16

**Authors:** Tongchao Xia, Min Hu, Chunmei Luo, Mengmeng Yang, Mingming Chu, Xing Xiang, Zhe Zhang

**Affiliations:** Department of Pharmacy, The Second Affiliated Hospital of Army Medical University, Chongqing, China

**Keywords:** analytic hierarchy process, anti-tumour, commercial Chinese polyherbal preparation, Delphi method, value evaluation system

## Abstract

**Objective:**

To develop a value assessment index system of anti-tumour commercial Chinese polyherbal preparation (CCPP).

**Methods:**

A modified two-round Delphi method was conducted to establish consensus within a field to reach an agreement via questionnaire among a multidisciplinary panel of experts. In addition, the analytic hierarchy process was used to conduct weight analysis of indicators.

**Results:**

In two rounds of Delphi consultation, the experts’ positive coefficient was over 90%, and the authoritative coefficient was over 0.70. The Kendall’s W of two rounds was 0.401 and 0.438, and the *P*-value of Kendall’s W test was all <0.001 for each round. The final value assessment index system consisted of 7 primary indicators, 24 secondary indicators, and 50 tertiary indicators.

**Conclusion:**

This value assessment index system could promote the clinical rational use of CCPP and the development of the drug catalog and clinical guideline in China.

## Introduction

Traditional Chinese Medicine (TCM), with a history spanning over five millennia in China, complements modern medicine through its unique theoretical system by integrating prevention, healthcare, treatment, and rehabilitation to enhance public health. China has published several policy documents, including the “Essentials of the Development Strategy for TCM(2016–2030)”, to promote TCM’s sustainable development and enhance its global recognition and acceptance (The State Council of the People’s Republic of China, 2016). Over centuries, TCM has become the most complete, influential, and widely used traditional medical system in the world today, which has spread to 193 countries and areas, including the United States, the European Union, Africa, and Southeast Asia through trade routes and modern globalization (Yagüe et al., 2022; Kadier et al., 2025). Based on TCM’s theory, commercial Chinese polyherbal preparation (CCPP) refers to Chinese medicinal products, which have been manufactured into pills, powders, granules, capsules, tablets, etc. It features convenient administration and storage, along with relatively low cost. Certain CCPP have been registered either as prescription drugs or over-the-counter medications in Russia, Canada, and the European Union, such as Huatuo Zaizao pill, Danshen Capsule, and Xiaoyao Pian (Zhang B. et al., 2022).

According to the latest data from the International Agency for Research on Cancer, projections estimate that by 2050, the annual number of new cancer cases will increase by 77% compared to 2022 levels (Bray et al., 2024). Cancer treatment faces long-term challenges, including inequitable healthcare resource allocation, high treatment costs, and compromised quality of life (Schütte et al., 2021; Bizuayehu et al., 2024). TCM demonstrates unique advantages as an integral part of comprehensive cancer treatment, which include improving patients’ quality of life, reducing risks of recurrence and metastasis, and prolonging progression-free survival, through modulation of the immune microenvironment and alleviation of radiotherapy- and chemotherapy-induced adverse reactions (Zhang et al., 2021; Xi et al., 2025; Gao et al., 2021).

However, several concerns have emerged regarding the increasing clinical utilisation rate of anti-tumour CCPP (Wu et al., 2022; Wang et al., 2024), including a large number of specifications, inconsistent drug standards, uneven drug quality, and the lack of standardisation in selection mechanisms and post-marketing evaluations (Chen et al., 2023). These result in insufficient reliable evidence for the inclusion of CCPP in critical decision-making tools within the clinical practice guidelines.

Relevant administrative departments in China have successively published multiple evaluation guidelines, based on the Health Technology Assessment (HTA) and Value-Based Healthcare over the years (Zhen et al., 2018; Guo et al., 2023), which focus on the value of health and healthcare interventions (including drugs, devices, diagnosis, and treatment plans). Previous studies developed an HTA-based methodology to develop value evaluation of drugs (Maxion-Bergemann et al., 2006; Zhang C. et al., 2022; Su et al., 2024). However, there are currently no validated, quantitative frameworks to guide CCPP value evaluation. Therefore, this study aimed to construct a value assessment index system of anti-tumour CCPP, which optimised the allocation of health resources, enhanced rational clinical utilisation of anti-tumour CCPP, and provided a reference for selecting anti-tumour CCPP.

## Methods

### Literature review and construction of preliminary indicator pool

A systematic literature review was conducted to develop an initial indicator pool. The search included Web of Science, PubMed, Embase, Cochrane Library, CNKI, Wanfang, VIP, and related professional websites, which lasted until September 2023. The related websites included National Comprehensive Cancer Network, American Society of Clinical Oncology, China Anti-cancer Association, and Chinese Society of Clinical Oncology. We used the following Chinese and English search terms: cancer, CCPP, drug, Health Technology Assessment, value framework, value evaluation. Based on the literature review, we held a small-scale expert consultation meeting to discuss the importance and suitability of the indicators. None of these experts participated in the follow-up Delphi expert consultation.

**FIGURE 1:**
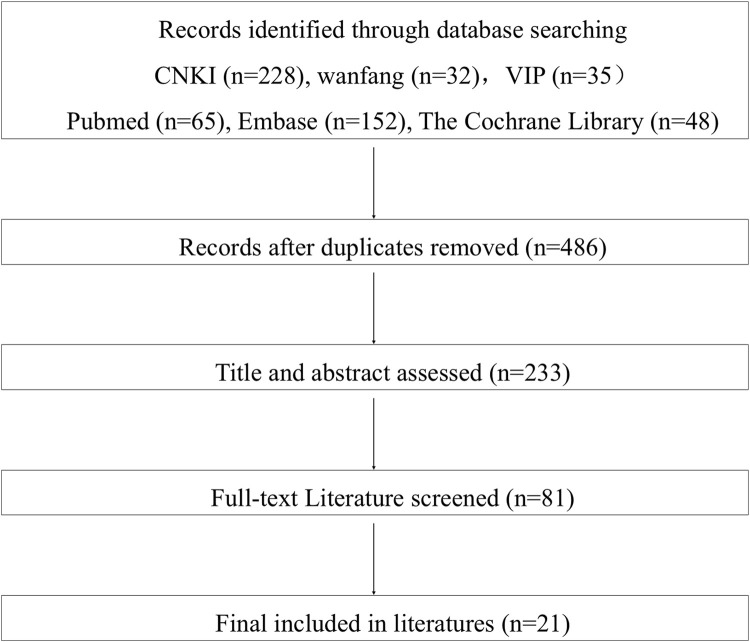
The flow chart of initial indicator construction.

The flow chart of the literature search is shown in Figure 1, and the 21 studies were finally included in the review (Supplementary Table S1). Finally, a preliminary indicator pool of value assessment of anti-tumour CCPP was developed, consisting of 7 first-level indicators, 26 second-level indicators, and 62 third-level indicators.

### Expert selection

We recruited 32 consulting experts nationwide, including clinical medicine, clinical pharmacy, pharmacy administration, evidence-based medicine, and pharmacoeconomics. The inclusion criteria for Delphi consultation experts were as follows: (1) being familiar with the research topic; (2) intermediate or higher professional title, and ensure that the proportion of experts with associate senior or higher professional titles is greater than 50%; (3) having >5 years of working experience in their field, and ensure that more than 50% of experts have more than 10 years of work experience.

### Data collection

The expert questionnaire included three parts: (1) introduction of the background and purpose of the study; (2) basic information of the experts: including title, years of experience, and research field, etc. (3) the expert consultation content: the framework of the three-level indicators, the experts rated the importance and operability of the indicators with Likert scoring method (Ruan et al., 2024). The experts’ familiarity scale with indicators ranging from 1 (very unfamiliar) to 5 (very familiar), and the experts’ basis of judgement included theoretical analysis, practical experience, peer understanding, and individual intuition.

This study completed 2 rounds of expert consultation using the modified Delphi method, with a return time of 2 weeks. Indicators included in the analysis had an average importance score ≥3.5 and the coefficient of variation (CV) ≤ 0.25 (Hommel et al., 2016), and the indicators would be revised, deleted, or added following a deliberation undertaken by the research team, when the experts suggested. Combining the results of the expert questionnaires and group discussion, the index system was finally determined.

### Data analysis

SPSS 22.0 was used to analyse and process the data. The coefficient of the authority (Cr) of the experts is the arithmetic average of the judgment coefficients (Ca) and the familiarity coefficients (Cs). In addition, the coefficient of the coordination of the experts’ opinions (Kendall’s W) indicated the degree of coordination of the experts’ opinions, that is, W = 12∗ $\sum_{i=1}^{n}d_{i}^{2}$ / $m^{2}\left(n^{3}‐n\right)$ . Where “m” is the total number of experts, “n” is the number of lists, and “di” is the value of the evaluation levels of “m” participants for the “i” lists minus the arithmetic average of the evaluation levels for all criteria.

Then, we determined the weights of the dimensions and indicators by the Analytic Hierarchy Process (AHP). First, we constructed a judgment matrix to compare indicators two by two using Saaty’s 1-9 scale. Second, the weights for each level of indicators were calculated. Third, the consistency ratio (CR) was used to test the logical consistency of the judgement matrix. The CR < 0.10 was considered acceptable, indicating that the pairwise comparisons are sufficiently consistent. At last, the weight for each sub-criterion was calculated by multiplying its local weight by the weight of its parent criterion in the level above, and then aggregating these results across the hierarchy.

## Results

### Characteristics of the experts

A total of 32 experts from various fields were selected to participate in this survey, whose research fields were mainly related to clinical pharmacy (10, 31.25%), clinical medicine (10, 31.25%), evidence-based medicine (4, 12.50%), pharmacy management (5, 15.62%), and pharmacoeconomics (3, 9.38%). The educational background of the specialists varied, with 18 (56.25%) holding a PhD, 12 (37.50%) holding a master’s degree. Of the participants, 19 (59.38%) held a senior associate position. The selected experts came from 11 provinces and cities, including Beijing, Shanghai, Chongqing, Sichuan, Jiangsu, Fujian, etc., with good geographical representation. More information about the expert group is shown in Table 1.

**TABLE 1:** General information of experts.

Category	Characteristics	Number	Percentage (%)
Gender	Male	14	44
	Female	18	56
Age	30∼39	16	50
	40∼49	14	44
	≥50	2	6
Educational attainment	Doctor’s degree	18	56
	Master’s degree	12	38
	Bachelor’s degree	2	6
Professional title	Senior	6	19
	Senior associate	19	59
	Middle	7	22
Main research areas	Clinical pharmacy	10	31
	Clinical medicine	10	31
	Evidence-based medicine	4	13
	Pharmacy management	5	16
	Pharmacoeconomics	3	9
Working years	5∼10	11	34
	11∼15	12	38
	≥16	9	28
Province or region	Chongqing	17	53
	Beijing	3	10
	Shanghai	1	3
	Tianjin	1	3
	Sichuan	3	10
	Yunnan	2	6
	Guizhou	1	3
	Fujian	1	3
	Jiangsu	1	3
	Jiangxi	1	3
	Guangxi	1	3

### Experts’ motivation and authority coefficient

In two rounds of the Delphi survey, 32 experts completed the questionnaire (100%), which demonstrates experts’ high motivation. The coefficient of the authority of the experts (Cr) was 0.84, which is higher than 0.70, indicating the reliability of consulting outcomes (Table 2).

**TABLE 2:** The authority coefficient of consulting experts.

Familiarity (Cs)	Judgment basis (Ca)	Authority coefficient (Cr)
0.81	0.86	0.84

### Consistency and coordination of experts’ opinions

In the first round, the mean importance score of each indicator ranged from 3.60 to 4.94, and CV ranged from 5.00% to 25.65%. In the second round, the mean importance score of each indicator ranged from 3.88 to 4.96, and CV ranged from 3.88% to 22.94%. The Kendall’s W coefficient was 0.401 and 0.438, suggesting that the consistency of experts’ opinions was acceptable (Table 3).

**TABLE 3:** Consistency coefficient of expert comments in two rounds of consultations.

Round	CV	Kendall’sW	χ^2^	P-value
1	0.16	0.401	76.995	<0.001
2	0.14	0.438	68.275	<0.001

### Indicator screening

Based on the first round of expert consultation, two secondary indicators were deleted (drug interactions, incremental cost-effectiveness ratio), and eleven tertiary indicators were deleted (defined daily doses, median disease-free survival, median duration of response, distant metastasis free survival, time to failure, time to progress, numbers of pharmaceutical enterprises, numbers of pharmaceutical circulation enterprise, off-label use, interactions with chemical drugs, medical staff medication experience). After the two rounds of expert consultations, the final value assessment index system of anti-tumour CCPP included 7 primary indicators, 24 secondary indicators, and 50 tertiary indicators.

### Final indicator weights

The results of the weight calculation indicated that the weights of primary indicators were 0.3341 (safety), 0.2880 (efficacy), 0.0792 (economics), 0.0849 (appropriateness), 0.0469 (innovation), 0.1089 (accessibility), and 0.0661 (Characteristics of Chinese medicine). And the consistency ratio value of every matrix’s consistent ratio was <0.1, which met the consistency verification requirements (Supplementary Table S2).

## Discussion

### Main findings

This research innovatively constructed a system for multiple dimensions of comprehensive assessment of anti-tumour CCPP of clinical application based on this realistic background, which shows significant practical value. First, the system established a value evaluation framework of dimensions, including pharmacoeconomics, clinical efficacy, safety, and characteristics of TCM, which provided a standardized decision-making tool for the entry of drugs into medical institutions. This structured evaluation model can effectively enhance the scientificity and standardization of selection for anti-tumour CCPP. Second, the evaluation system can serve multiple dimensions setting of the drug Administration setting. It can provide a quantitative basis for the substitution of drug varieties in hospitals as well as evidence-based support for the formulation of drug directories by government departments.

### Strengths and limitations

Firstly, we invited 32 experts from 11 provinces and cities in China to form an expert group, adopting the Delphi method. The experts’ research directions involved seven specialised fields, including clinical medicine (oncology and TCM-related), clinical pharmacy (oncology and TCM-related), evidence-based medicine, pharmaceutical administration, and pharmacoeconomics. The experts, with 78.13% serving in senior professional titles, and 87.50% having more than 10 years of work experience, indicate that the selected experts have good professionalism and authority. The recovery rate of the consultation forms for both rounds of consultation is 100%, and the recovery time is basically around 2 weeks, indicating that the experts have good enthusiasm. The Cr value stands for the degree of expert authority, which is a higher authority when it is more than 0.7. After two rounds of expert consultation, Cr is 0.84, which demonstrates the superior authority of the experts and credibility of the research results. In this research, the Kendall’s W coefficient of expert opinion coordination was 0.401 and 0.438, respectively, and *P* < 0.001 during the comparison of two rounds. It indicates the high consistency of expert opinions and the stability of evaluation results.

Secondly, in order to emphasise the characteristics and advantages of CCPP, the first-level indicator (Characteristics of Chinese medicine) was specifically added to the value assessment index system, with all experts agreeing.

Thirdly, a reasonable weight setting is crucial for establishing an index system. The combined dominance of the “Safety” and “Efficacy” criteria (over 60%) indicates a strong consensus among experts that safety and efficacy are the fundamental prerequisites for further value consideration. It suggests that in a multi-criteria decision analysis framework, a poor performance on either of these criteria could act as a potential veto, even if the technology scores highly on other aspects like cost-effectiveness or innovation. This validates the structure of our criteria framework, confirming that the panel prioritised the core requirements for regulatory approval and clinical adoption. The safety and efficacy of our weighting scheme show strong alignment with the core evaluation pillars of major international HTA bodies. Such as the UK’s National Institute for Health and Care Excellence and Canadian Agency for Drugs and Technologies in Health, which base their assessments fundamentally on clinical effectiveness (encompassing efficacy) and safety.

Fourthly, we collected experts’ feedback during each round to gain a deeper understanding of indicators, as well as ensuing guidance for satisfactory practice, so that the index system could be more appropriate for value assessment.

However, the study also has some limitations. First of all, no expert centralised discussions were organised to address different viewpoints, which made it more difficult to reach a consensus, resulting in insufficient consistency of results. In future research, we suggest that implementing sensitivity analysis could more effectively address the complexities and uncertainties involved in evaluating the value of anti-tumour CCPP. Secondly, a formal content validity study before the Delphi rounds would have further strengthened the initial questionnaire. Thirdly, the weight assigned to the Characteristics of Chinese medicine was 0.0661, which is significantly lower than that for safety and efficacy. This discrepancy may stem from the limited representation of Chinese medicine experts among the Delphi panellists in this study. Fourthly, the absence of patient or policymaker perspectives may limit comprehensiveness, although we aim to develop an assessment tool for value evaluation of anti-tumour CCPP from the medical institutions perspective, which can provide a basis for hospital drug access decisions and clinical medication recommendations. The perspectives of patients or policymakers will be considered for inclusion in the evaluation system. In subsequent research, the reliability and validity of the anti-tumour CCPP evaluation system will be verified using selected anti-tumour CCPPs, which are selected from the national drug directory.

### Applications in research and clinical practice

The development of the value assessment index system is not merely an academic exercise but provides a foundational instrument with significant potential to guide future research and enhance real-world clinical decision-making. Its applications can be explored in several key directions: ⅰ) The tool’s structured criteria can help identify specific evidence deficiencies for individual drugs, particularly regarding comparative effectiveness, safety in real-world settings, and pharmacoeconomic value. For instance, for a CCPP scoring high on traditional evidence but low on modern pharmacological mechanism validation, future research can be directed towards employing advanced techniques like bioinformatics and molecular biology to elucidate its pathways and potential drug targets. ⅱ) In clinical practice, a validated value assessment index system can be integrated into Clinical Decision Support systems to provide clinicians with a standardised, evidence-based evaluation of CCPP. ⅲ) The tool offers a transparent methodology for drug formulary reviews and health technology assessment. Its structured evaluation of safety, efficacy, and especially economic value can provide critical data to inform reimbursement decisions by national insurance schemes and guide the inclusion of CCPP into the essential drug list and clinical practice guideline.

## Conclusion

In summary, this study developed a value evaluation system of anti-tumour CCPP through two rounds of Delphi expert consultation and the analytic hierarchy process. The system encompasses 7 first-level indicators, 24 second-level indicators, and 50 third-level indicators. And we confirmed that the value assessment index system could provide a reference to promoting the clinical rational use of CCPP and the development of the clinical guideline.

## Data Availability

The original contributions presented in the study are included in the article/Supplementary Material, further inquiries can be directed to the corresponding author.
