# Supplementary material for: Developing a value assessment index system of anti-tumour commercial Chinese polyherbal preparation in China: a modified Delphi study
Source: Front Pharmacol. 2025 Dec 16;16:1681174. doi: 10.3389/fphar.2025.1681174 (PMC12747457; doi:10.3389/fphar.2025.1681174)
Supplement: Supplementary file 1 [file DataSheet1.pdf]

SUPPLEMENTAL FILE

Title : Developing a value assessment index system of anti-tumour commercial Chinese polyherbal preparation in China: a modified Delphi study

Table S1 The studies finally included in the Literature review

Number	Title	Author	Journal/Website	Year
1	National Comprehensive Cancer Network. NCCN clinical practice guidelines in oncology (NCCN Guidelines) with NCCN evidence blocks	National Comprehensive Cancer Network	https://www.nccn.org/evidenceblocks/default.aspx	2021
2	A case of multi-dimensional value judgment and evaluation toolkit of pharmaceuticals	Guo et al.	Health Development and Policy Research	2020
3	Technical Specification for Clinical Comprehensive Evaluation of Chinese Patent Medicine	Zhang et al.	World Chinese Medicine	2021
4	Establishment of Drug Value Indicators System	Lin et al.	China Pharmacy	2013
5	The research on the value framework of Chinese patent medicine	Dang et al.	Chinese Journal of New Drugs	2021
6	American Society of Clinical Oncology Statement: A Conceptual Framework to Assess the Value of Cancer Treatment Options	Schnipper LE et al.	Journal of Clinical Oncology	2015
7	A standardised, generic, validated approach to stratify the magnitude of clinical benefit that can be anticipated from anti-cancer therapies: the European Society for Medical Oncology Magnitude of Clinical Benefit Scale (ESMO-MCBS)	Cherny NI et al.	Annals of Oncology	2017
8	Guideline for clinical comprehensive evaluation of Chinese patent medicine (2022 version)	Yuan et al.	China Journal of Chinese Materia Medica	2023
9	International standards for health economic evaluation with a focus on the German approach	Riedel R et al.	Journal of Clinical Pharmacy and Therapeutics	2013
10	Overview of the ICER value assessment framework and update for 2017–2019	Institute for Clinical and Economic Review	https://icer.org/wp-content/uploads/2017/06/ICER-value-	2020

			assessment-framework-Updated-050818.pdf	
11	Advances in Drug Market Value Evaluation Methods Research	You et al.	Chinese Journal of Pharmacoepidemiology	2016
12	Construction Study of Index System to Evaluate the Clinical Value of Drugs	Zhang et al.	Chinese Pharmaceutical Journal	2017
13	Study on Evaluation Indicator System of Drug Accessibility	Gong et al.	Chinese Health Economics	2011
14	Key considerations and issues of assessment for clinical value of drugs	Liu et al.	Chinese Journal of New Drugs	2017
15	Thoughts on foreign universal drug value evaluation tools for construction of Chinese patent medicine value evaluation tools	Zhang et al.	China Journal of Chinese Materia Medica	2021
16	Thoughts on post-marketing evaluation of classical Chinese patent medicines based on clinical value	Song et al.	China Journal of Chinese Materia Medica	2021
17	Research on core assessment indicators for human use experience of traditional Chinese medicine based on Delphi method	Zhi et al.	Chinese Journal of New Drugs	2023
18	Expert consensus on core indicators for lifecycle value assessment of Chinese patent medicine	Yu et al.	China Journal of Chinese Materia Medica	2023
19	Guideline for multi-dimensional and multi-criteria comprehensive evaluation of Chinese patent medicine	Institute of Basic Research in Clinical Medicine	Chinese Journal of Evidence-Based Medicine	2022
20	Exploration and practice of post-marketing survival benefit evaluation of Chinese patent medicine for cancer	Xu et al.	China Journal of Chinese Materia Medica	2021
21	Methods of developing core traditional Chinese medicine syndromes set	Qiu et al.	Chinese Journal of Evidence-Based Medicine	2021

Table S2 The ealue evaluation indexs ystem of anti-tumor CCPP

Primary indicators	Weight	Secondary indicators	Combined weight	Tertiary indicators	Combined weight
Safety	0.3341	Incidence of adverse events	0.1177	Overall incidence of adverse events	0.0462
				Incidence of adverse events of grade 3 and above	0.0715
		Medication for special population	0.0551	Special requirements for medication in special populations	0.0551
		Drug toxicity	0.1110	toxic raw Chinese medicines in formula	0.1110
		Government control	0.0503	Whether the drug administration has issued warnings, withdrawals and recalls	0.0270
				Whether the drug administration has issued notification information on drug safety	0.0098
				Modification of drug instructions due to safety	0.0136
Efficacy	0.2800	Main clinical indicators	0.1070	Median overall survival	0.0727
				Median progression-free survival (PFS)	0.0343
		Minor clinical indicators	0.0308	Objective response rate	0.0308
		Core TCM syndromes	0.0624	Core traditional Chinese medicine syndromes (Evaluation criteria of TCM clinical syndrome score)	0.0624
		Patient-Reported Outcomes	0.0490	Score criterion of Quality of Life (QOL).	0.0490
		Recommended level of evidence	0.0309	Disease diagnosis and treatment norms, clinical guidelines, expert consensus recommendations	0.0108

Economics	0.0792	Drug treatment costs	0.0792	Recommended level and evidence strength	0.0201
				Daily drug cost (DDC)	0.0328
				Single course drug cost	0.0464
Suitability	0.0849	Technical suitability	0.0222	Clear indication population of instructions	0.0075
				Completeness of instruction information and labels	0.0056
				Drug storage and transportation conditions	0.0020
		Suitability for use	0.0235	Drug monitoring or follow-up	0.0021
				Treatment of adverse drug reactions	0.0050
				Consistency of usage and dosage, route and interval of administration with the instructions	0.0162
				Patient medication experience	0.0074
		Maturity of drugs	0.0236	Medical Insurance Catalog	0.0094
				National essential medicine list	0.0088
				Protection of TCM species	0.0054
		Management compatibility	0.0155	National/Provincial Key Monitoring Drug Catalogue	0.0078
				Centralized procurement of traditional CCPP	0.0077
Innovation	0.0469	Clinical innovation	0.0225	Urgent clinical needs or blank varieties	0.0158
				Significant therapeutic advantages (bioavailability, efficacy, etc.)	0.0067
		Technological innovation	0.0140	Significant technological advantages (stability, dosage form, taste, etc.)	0.0140
		Industrial Innovation	0.0103	Pharmaceutical Patents (domestic / international)	0.0103

Accessibility	0.1089	Availability	0.0593	Drug shortage	0.0593
		Sustainability	0.0496	Sustainability of raw Chinese medicines	0.0496
Characteristics of chinese medicine	0.0661	Rationality of formula	0.0187	Theories of TCM	0.0085
				Pharmacological mechanism	0.0047
				Drug-syndrome compliance analysis	0.0055
		Drug-syndrome compliance	0.0171	Accuracy of target or functional indications	0.0109
				Integrity of pharmacological research on formula	0.0062
		Similarity of formula	0.0050	Similarity of formula composition and indications	0.0050
		Reliability of human experience	0.0111	Randomized controlled clinical trial data	0.0032
				Large-scale clinical observational trial data	0.0025
				Clinical system cases data	0.0015
				Open-labelor small sample clinical trials	0.0010
				Clinical-based big data and bioinformatics analysis	0.0011
				Related literature	0.0006
				Originating from famous Classical formula, Empirical or Ancient formula	0.0011
				Whether the Chinese Pharmacopoeia and departmental drug standards record	0.0068
		Quality controllability	0.0142	Integrity of quality standards	0.0031
				Toxic metabolite content	0.0043
